# Celibacy

**Published:** 1857-11

**Authors:** 


// HALL'S JOURNAL OF HEALTH.

OUR LEGITIMATE SCOPE IS ALMOST BOUNDLESS: FOR WHATEVER BEGETS PLEASURABLE AND HARMLESS FEELINGS, PROMOTES HEALTH; AND WHATEVER INDUCES DISAGREEABLE SENSATIONS, ENGENDERS DISEASE.

VOL. IV.] NOVEMBER, 1857. [NO. XI.

WE aim to show how disease may be avoided, and that it is best, when sickness comes, to take no medicine without consulting an educated physician.

### CELIBACY

Is the ruin of any nation; it is a greater moral curse than drunkenness ever has been; and the parent who countenances it is, to that extent, his child's worst enemy, whether that child be son or daughter. As nations and communities prosper and grow older, the lines between wealth and poverty become more distinct; and, with equal pace, the strife for riches becomes a passion and a desperation, with a declining morality: and with all this, fewer persons marry, and they, at a later age in life—the unmarried setting up private establishments; while the more successful inaugurate a style of living largely beyond the profits of their business; when on any financial jar their rickety plans are deranged, and they are the first to go into bankruptcy. But, besides these two classes, there is a third, far outnumbering both, whose profligacy is of the more common sort, spreading its demoralizing and corrupting influences far and wide. Look at Stockholm, the capital of Sweden, once the fear of nations, when Charles the Fifth led her armies! But now, enervated by her demoralizations, she exists only by sufferance. In Stockholm, very near *half* of the registered births are illegitimate. In Paris, one child in every three is born out of wedlock. It is very easy to see that the cause of this is largely owing to deferred marriage. In our own country, especially in our large cities, the fact is everywhere observed, that young men are putting off marriage till a later and later period of life. Very many do not entertain the thought of it until past thirty; in the mean time, as a matter of course, do worse—and they begin to complain of rheumatisms, the patent indicator of their immoralities.

But why do young men put off marriage until thirty and over? The reasons are twofold. Poverty and pride. They cannot afford to live in the style which their ambition aspires to. Young people want to begin at too high a round in the social ladder. Formerly, a young man was respected who married, and lived in the rear of his house, while the front was his office, or shop, or store; and when he got a little farther on, he occupied the first floor as his place of business, while his wife and children lived up stairs, *and it ought to be so still*, by three-fourths of the business men of our large cities. It is capable of demonstration, that it is healthier to do so, and a great economizer of time and money. To live "*up town*," for example, in New York, three or four miles away from a man's place of business, is neither as good for himself or family, soul, body, or estate, *in the long run*, as if he did business on the first floor, and his family lived on the second or third of the same building.

A business man is always anxious when away from his place of business. He has a peace of mind when on the spot which is of itself a treasure. Bear us witness of this ye business men, who live in streets above the thirties, or "*out of town.*"

By the old plan, men enjoyed their families, lived with their wives and children, ate at home always, always slept at home; and in the end, lived longer, happier, and died richer, and more honored, than do the same class of persons now-a-days, with their empty store-lofts down town, and their dwellings miles away, at an extra rent of from one to three thousand dollars a year.

Compare the hasty and unmasticated dinner, or the late repast of five P. M., all jaded, exhausted, depressed with ten hours' labor, with the cozy dinner at decent old-fashioned noon—just hungry enough to enjoy, just tired enough to make a half-hours' sitting grateful, and just exhilarated enough with the profits of the morning to make it a matter of pleasureable converse while eating.

We have our eye now on two Dutch shoemakers and a native grocer, who have been living in this old-fashioned style within a block of us, in another street, for several years, and every month or two, we notice some new indication of thrift, in a cleaner side-walk, a more showy window, a larger stock, or newly-painted front, and they are already able to own the houses they live in!—are saving something every year.

The above was written some three months ago; and what a confirmation of its truth is now found in the commercial panic of the middle of October, eighteen hundred and fifty-seven! Just look at it! Within a week, there appeared, in the same day's paper, an item, that one merchant, who stood up in the crisis of "*thirty-seven*," failed for the want of seven thousand dollars; another, whose house was deemed impregnable, and who could have raised in the street, in any business hour, a hundred thousand dollars on his own name, sixty days ago, *failed* for the want of six thousand dollars! Deduct the price of an up-town house, say twenty-five thousand dollars, and how many a man would most triumphantly have weathered this financial tornado! Deduct twenty-five hundred dollars rent, with its accumulated interest for ten years past, and how many a young merchant of New York would have to day walked the street with a firmer tread and a prouder look, who now, with an overdrawn bank-account, a bowed head, and broken ambition, has but one absorbing wish—to be shut out from the sight and memory of men for a season; or, that they could be so happy, as to be able to begin the world again without a dollar of capital, and without a dollar of debt.

Young men, you who are to be our merchant princes twenty years hence, think of this! *Marry early*, LIVE UNOSTENTATIOUSLY, AND RETIRE WITH A MODERATE FORTUNE.

---

### STRANGE!

A YOUNG lady crossing over from Jersey the other day, is stated, on good authority, to have courteously thanked a gentleman who gave her his seat!

---

### TRUE.

THE man who drew a conclusion, has since died of the effort.

